# Posterior nasal hemorrhage mimicking intra-abdominal hemorrhage after gastrectomy: a case report

**DOI:** 10.3389/fsurg.2026.1793381

**Published:** 2026-05-29

**Authors:** Haixuan Ye, Yan Huang, Zexian Xu, Huanshen Huang, Tianyu Qiu, Jiajia Chen

**Affiliations:** 1 Department of Hepatobiliary Surgery, Chaozhou Central Hospital, Chaozhou, China; 2 Department of the First Clinical Medical College, Southern Medical University, Guangzhou, China

**Keywords:** anastomotic leak, drain migration, gastric cancer, posterior nasal hemorrhage, postoperative hemorrhage

## Abstract

**Background:**

Postoperative hemorrhage is a serious complication following gastrectomy, most commonly resulting from anastomotic bleeding or pseudoaneurysm rupture. When conventional diagnostic modalities fail to identify the source of bleeding, establishing an accurate diagnosis can be extremely challenging.

**Case presentation:**

We report the case of a 77-year-old Asian woman diagnosed with gastric malignancy who underwent Roux-en-Y total gastrectomy in our department. On postoperative day 6, she developed an esophagojejunal anastomotic leak. On postoperative day 28, the patient experienced hemorrhage, with bright red blood draining from the abdominal drain, mimicking delayed extra-intestinal bleeding. However, abdominal computed tomography (CT), endoscopy, and digital subtraction angiography (DSA) revealed no evidence of intra-abdominal hemorrhage. Endoscopic evaluation further demonstrated that the abdominal drain had migrated into the jejunal lumen through the anastomotic leak. Ultimately, nasendoscopy identified the true source of bleeding: the posterior nasal cavity. Blood from this site entered the digestive tract via the pharynx and was externally drained through the displaced abdominal drain. Hemostasis was successfully achieved using endoscopic electrocautery.

**Conclusion:**

In cases of post-gastrectomy hemorrhage where contrast-enhanced CT, endoscopy, and DSA fail to localize the bleeding source, clinicians should actively investigate for remote or occult bleeding sites and consider atypical drainage pathways.

## 1 Introduction

Postoperative hemorrhage is the most serious complication following radical gastrectomy. Reported incidence rates range from 1% to 4%, with associated mortality as high as 10%–20%. In cases caused by pseudoaneurysm rupture, the mortality rate may reach up to 50% (1, 2). Post-gastrectomy hemorrhage can be classified according to timing, early or delayed, and by location, intra-intestinal or extra-intestinal (3). Delayed bleeding, defined as hemorrhage occurring more than seven days after surgery, typically presents as extra-intestinal bleeding and is most often caused by pseudoaneurysm rupture or vascular erosion (3). For the diagnosis of post-gastrectomy hemorrhage, contrast-enhanced CT allows rapid localization of intra-abdominal bleeding, digital subtraction angiography (DSA) can precisely identify the bleeding vessel and facilitate simultaneous embolization, and endoscopy enables direct visualization of intraluminal bleeding sites (4).

Anastomotic leak is a serious complication following radical gastrectomy. Reported incidence rates range from 1.2% to 6.7% (5, 6), and its occurrence is influenced by multiple factors, including advanced age, comorbidities, nutritional status, and surgical technique (7). Leakage of digestive fluids, such as gastric, pancreatic, or intestinal secretions, can erode adjacent blood vessels, potentially resulting in pseudoaneurysm formation or anastomotic bleeding (3). Consequently, in patients with an anastomotic leak who present with delayed extra-intestinal hemorrhage, a high index of suspicion for anastomotic bleeding or pseudoaneurysm rupture is warranted.

Although contrast-enhanced CT, endoscopy, and DSA are valuable tools for diagnosing post-gastrectomy hemorrhage, rare cases may occur in which the bleeding source remains undetected by these conventional modalities. Here, we report an unusual case in which posterior nasal cavity bleeding entered the digestive tract via the pharynx and was subsequently drained externally through an abdominal drain that had migrated into the jejunum through an anastomotic leak, thereby mimicking the clinical presentation of delayed extra-intestinal hemorrhage.

## 2 Case description

### 2.1 Preoperative and intraoperative findings

A 77-year-old Asian woman was admitted to our department with gastric cardia adenocarcinoma diagnosed by gastroscopy. She had no history of peptic ulcer disease, liver cirrhosis, hematologic disorders, recurrent epistaxis, or prior nasal surgery, and denied tobacco and alcohol use. There was no family history of gastrointestinal malignancy. On admission, her vital signs were stable, and no significant abnormalities were identified on general or abdominal examination. Preoperative laboratory investigations revealed a hemoglobin level of 112 g/L, with normal white blood cell and platelet counts, as well as normal coagulation, liver, and renal function. Contrast-enhanced abdominal CT demonstrated localized thickening of the mucosa along the posterior wall of the gastric fundus and cardia, with clear demarcation from the surrounding normal gastric wall and no evidence of regional lymphadenopathy, corresponding to a clinical stage of cT2N0M0 (stage IIA). Following multidisciplinary evaluation, neoadjuvant therapy was not recommended. One week after admission, the patient underwent laparoscopic total gastrectomy with D2 lymphadenectomy and Roux-en-Y esophagojejunostomy. Intraoperatively, bilateral abdominal drains were placed adjacent to the esophagojejunal anastomosis, along with a nasojejunal feeding tube and a nasogastric tube. Estimated intraoperative blood loss was approximately 50 mL, and the operative time was 5 h. Postoperative histopathological examination revealed a moderately differentiated tubular adenocarcinoma involving the gastric fundus and cardia, with invasion through the full thickness of the gastric wall into the extraserosal adipose tissue. No lymphovascular invasion, perineural invasion, or lymph node metastasis was identified. Immunohistochemical analysis showed p53 (mutant type), Ki-67 (90%), CK7 (+), CK20 (+), CEA (+++), HER2 (0), MLH1 (+), MSH2 (+), MSH6 (+), and PMS2 (+).

### 2.2 Postoperative anastomotic leak and management

Postoperatively, the patient received prophylactic antibiotics, acid suppression, fluid resuscitation, and other routine supportive treatments. On postoperative day 1, laboratory tests showed a white blood cell count of 12 × 10^9^/L, C-reactive protein of 80 mg/L, hemoglobin of 107 g/L, and albumin of 29 g/L; albumin supplementation was administered accordingly.

On postoperative day 6, the patient developed sudden, persistent upper abdominal pain, and approximately 200 mL of bilious fluid was drained from the abdominal drain. Physical examination revealed abdominal wall rigidity, localized upper abdominal tenderness, mild rebound tenderness, and decreased bowel sounds. Repeat laboratory tests demonstrated a white blood cell count of 15 × 10⁹/L, C-reactive protein of 120 mg/L, hemoglobin of 95 g/L, and albumin of 32 g/L. Abdominal CT showed fluid accumulation around the esophagojejunal anastomosis and a small amount of pneumoperitoneum, leading to a diagnosis of anastomotic leak. In addition, CT imaging demonstrated that the tip of the left-sided abdominal drain was located near the diaphragmatic level; however, it was unclear whether it had entered the gastrointestinal tract. Conservative management was initiated, including strict fasting, abdominal irrigation, continuous negative-pressure drainage, total parenteral nutrition, and empirical antibiotic therapy.

Between postoperative days 6 and 12, the patient remained nil per os. Daily drainage output decreased from 600 mL to 200 mL. The white blood cell count and C-reactive protein levels gradually declined, hemoglobin remained stable between 90 and 110 g/L, and albumin levels ranged from 30 to 32 g/L. Abdominal pain progressively improved, and there was no worsening of peritoneal signs.

On postoperative day 13, upper abdominal pain had largely resolved, with no abdominal tenderness and restoration of bowel sounds. Follow-up abdominal CT demonstrated a reduction in perianastomotic fluid, resolution of pneumoperitoneum, and no change in drain position.

From postoperative days 13 to 22, enteral nutrition was gradually initiated and advanced. Conservative management, including prophylactic antibiotics, acid suppression, and albumin supplementation, was continued. Laboratory tests repeated every 3–5 days showed that inflammatory markers had largely normalized, hemoglobin remained stable between 90 and 110 g/L, and albumin increased to approximately 40 g/L. Daily drainage output further decreased to 50–100 mL, with the character of the drainage fluid changing from bilious and turbid to clear, yellowish fluid. The patient reported no abdominal pain, fever, or other discomfort; vital signs remained stable, and there was no recurrence of peritoneal irritation.

On postoperative day 23, abdominal CT revealed near-complete resolution of fluid around the esophagojejunal anastomosis, with no change in drain position. However, an upper gastrointestinal barium study continued to demonstrate contrast extravasation at the anastomotic site, indicating that the leak had not yet healed. Conservative management was therefore continued.

### 2.3 Postoperative hemorrhage and diagnostic process

**Figure 1:**
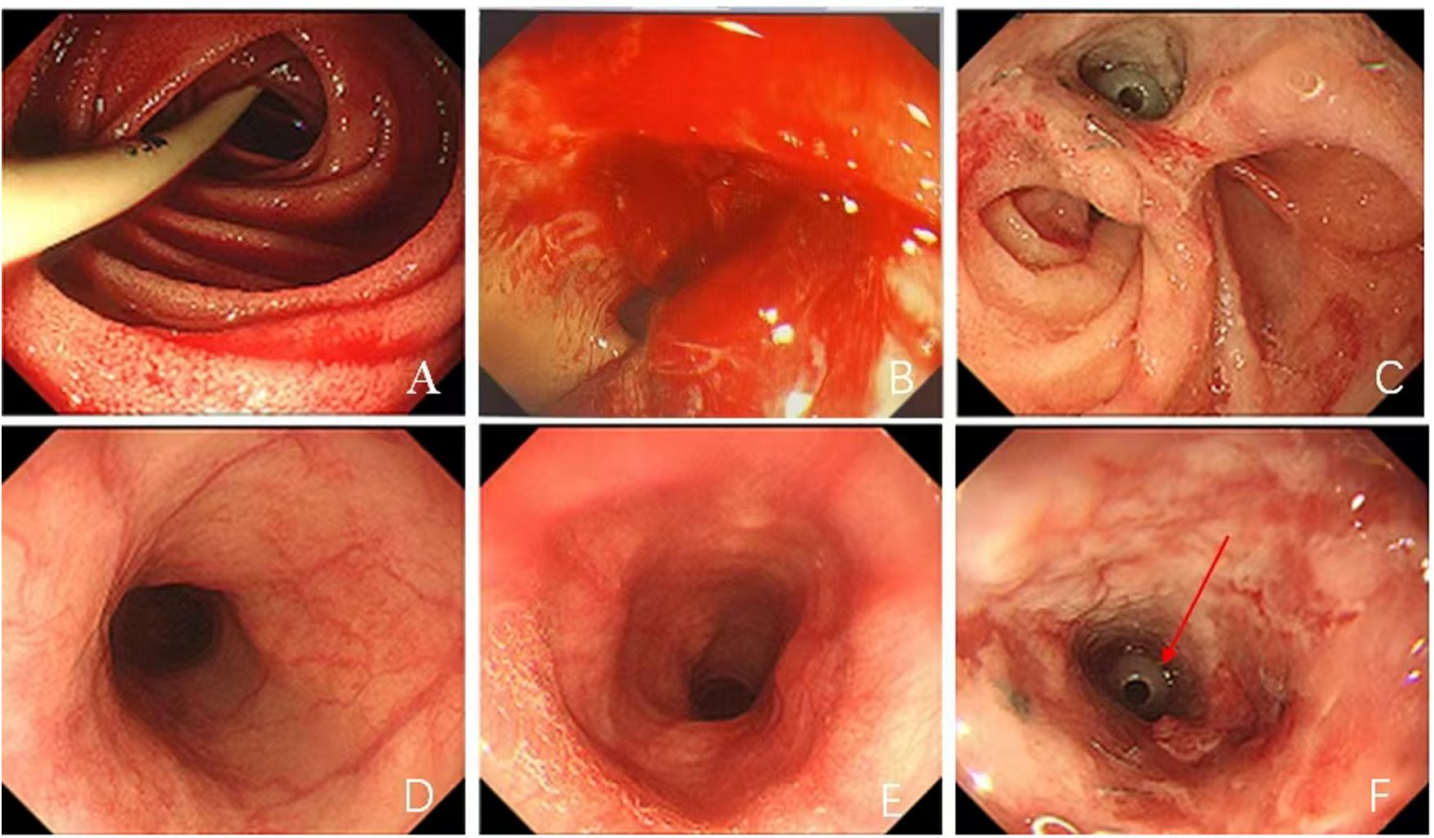
**(A,B)** A large amount of residual blood in the jejunum; **(C–E)** No bleeding at the anastomosis or in the esophagus; **(F)** abdominal drain migrated through the anastomotic fistula into the jejunum (arrow).

At 06:00 on postoperative day 28, approximately 200 mL of bright red blood was suddenly drained from the abdominal drain, accompanied by mild hemoptysis. No epistaxis was observed. On physical examination, the patient's blood pressure was 109/70 mmHg, heart rate was 88 beats/min, and there were no signs of abdominal rigidity or rebound tenderness. Conservative management, including acid suppression, hemostatic therapy, and blood transfusion, was initiated promptly. Repeat laboratory tests showed a hemoglobin level of 103 g/L with normal coagulation parameters. Emergency abdominal CT revealed no evidence of intra-abdominal hematoma. Urgent endoscopic examination demonstrated a large amount of residual blood within the jejunum (Figures 1A,B), but no active bleeding source was identified in the esophagus, anastomotic site, or jejunal mucosa (Figures 1C–E). Notably, endoscopy also revealed that the left-sided abdominal drain had migrated into the gastrointestinal tract through the anastomotic leak (Figure 1F), which may account for the delayed healing of the anastomosis. In the absence of an identifiable active intra-abdominal bleeding source and with no further episodes of bloody drainage, it was initially presumed that the hemorrhage had either ceased spontaneously or originated from an unvisualized site. Conservative management was therefore continued, with close monitoring of hemoglobin levels and clinical status.

**Figure 2:**
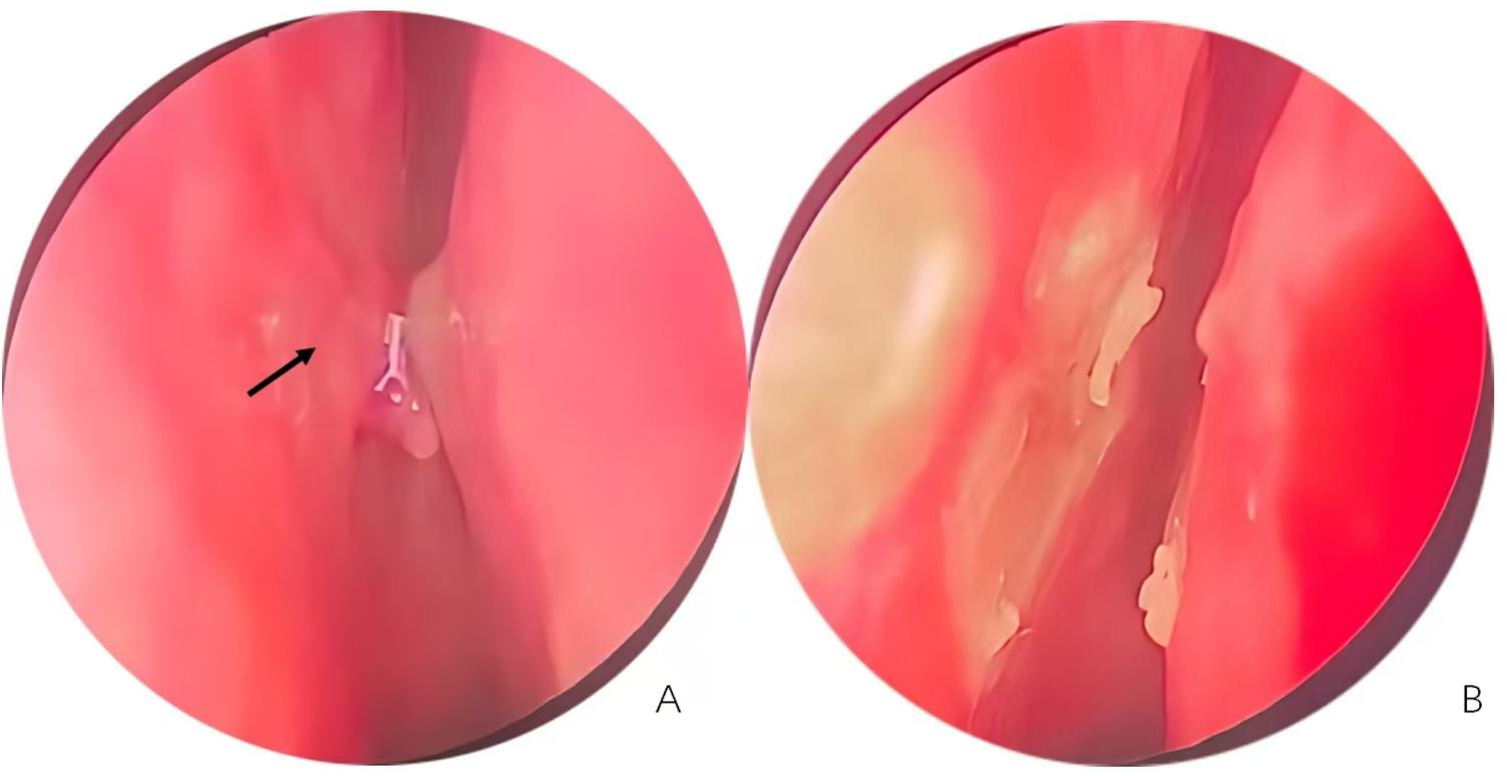
**(A)** Ruptured hemangioma with active bleeding in the posterior nasal cavity (arrow). **(B)** Status after endoscopic electrocoagulation for hemostasis.

**Figure 3:**
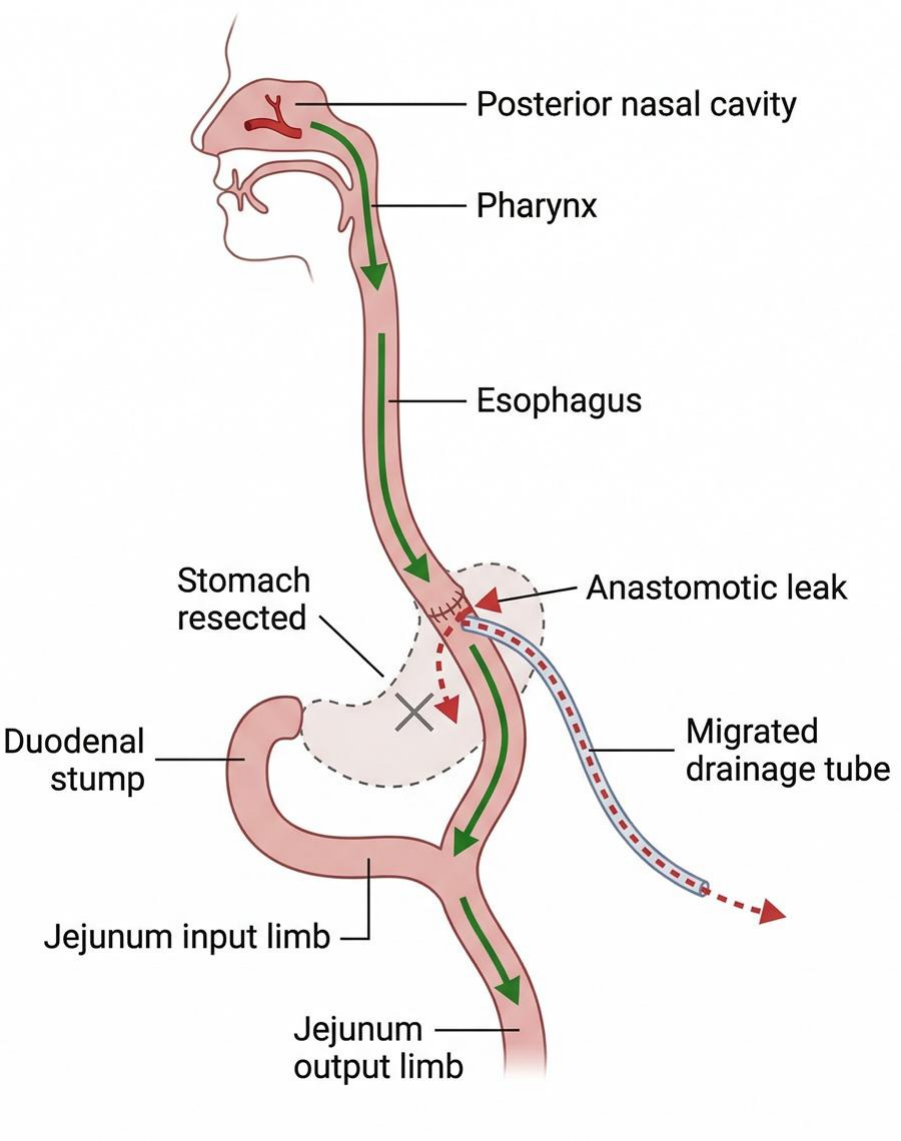
Posterior nasal cavity bleeding drains into the digestive tract and is aspirated out through the displaced drain via the anastomotic leak.

At 14:00 on postoperative day 28, approximately 500 mL of bright red blood was again drained from the abdominal drain, accompanied by recurrent hemoptysis. The patient's blood pressure decreased to 92/58 mmHg, heart rate increased to 112 beats/min, and hemoglobin dropped to 80 g/L. Given the recurrent bleeding of unclear origin, DSA of the celiac trunk and superior mesenteric artery was performed. However, no vascular abnormalities or contrast extravasation were identified. The patient was subsequently transferred to the intensive care unit for further management. At this stage, three key diagnostic modalities, CT, DSA, and endoscopy, had all failed to localize the source of bleeding, rendering the diagnosis particularly challenging. A multidisciplinary team was promptly convened, including specialists in gastroenterology, anesthesiology, radiology, and intensive care. A pivotal clue was provided by the anesthesiologist, who observed continuous fresh blood dripping from the patient's posterior nasal cavity during endotracheal intubation. In conjunction with the patient's hemoptysis, this finding raised suspicion of a respiratory tract source of bleeding. An emergency nasendoscopic examination performed by the otolaryngology team revealed an actively bleeding vascular lesion, resembling a hemangioma, in the posterior nasal cavity (Figure 2). Endoscopic electrocautery was successfully carried out to achieve hemostasis. Owing to the patient's critical condition, no biopsy of the lesion was obtained. The mechanism of hemorrhage was thereby clarified (Figure 3): bleeding from the posterior nasal cavity entered the gastrointestinal tract via the pharynx and was subsequently drained externally through the displaced abdominal drain via the anastomotic leak.

### 2.4 Subsequent management and outcomes

**Figure 4:**
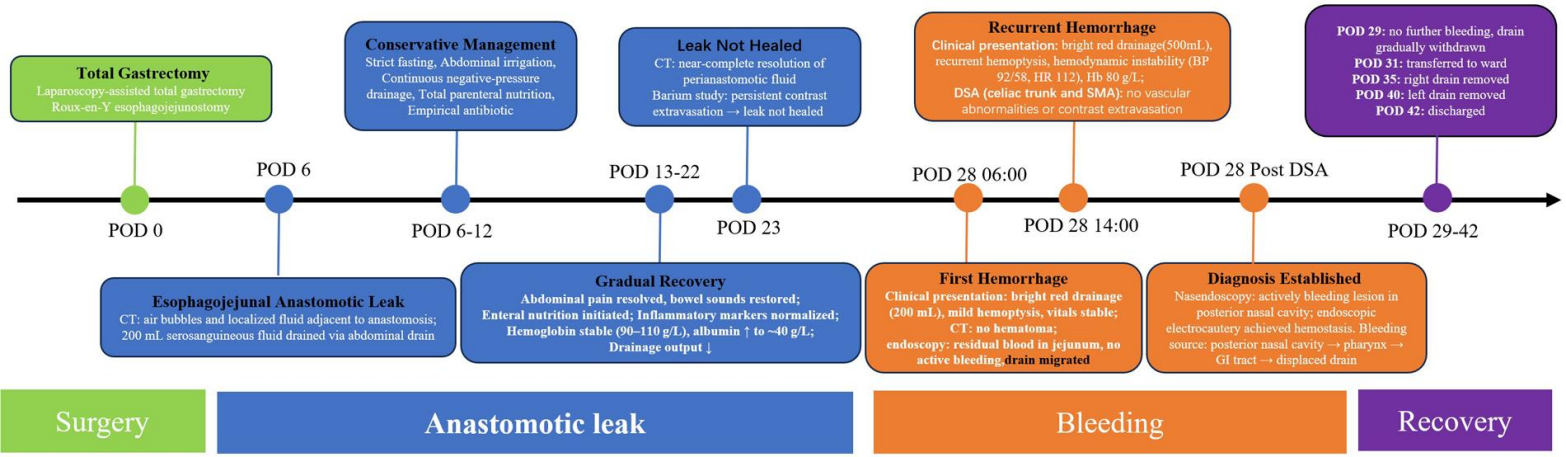
Clinical timeline illustrating the four phases of the patient's hospital course: surgery (POD 0), anastomotic leak (POD 6–28), hemorrhage and diagnostic workup (POD 28), and recovery (POD 29–42). POD, postoperative day; CT, computed tomography; DSA, digital subtraction angiography; GI, gastrointestinal.

On postoperative day 29, the patient remained in the intensive care unit. Only a small amount of serosanguineous fluid was noted from the abdominal drain, with no further episodes of bright red bleeding. The patient was alert, hemoptysis had resolved, and nasendoscopy demonstrated a clotted bleeding site in the posterior nasal cavity without evidence of active hemorrhage. Gradual withdrawal of the abdominal drain was initiated to facilitate healing of the anastomotic leak. By postoperative day 31, the patient's vital signs were stable, hemoglobin levels remained above 90 g/L, and the patient was transferred to the general ward. On postoperative day 35, abdominal CT revealed no fluid collection around the anastomosis, and the right-sided abdominal drain was removed. The left-sided drain was subsequently removed on postoperative day 40. The patient was discharged in stable condition on postoperative day 42. The patient declined adjuvant chemotherapy. At the six-month telephone follow-up, the patient was in good general condition, tolerating a normal diet, and had experienced no recurrence of bleeding. The clinical course is summarized in Figure 4.

## 3 Discussion

Delayed extra-intestinal hemorrhage is most commonly caused by pseudoaneurysm rupture or vascular erosion (1). In the present case, the presence of an anastomotic leak constituted a significant risk factor, and the clinical manifestations closely resembled those of delayed extra-intestinal hemorrhage. Accordingly, anastomotic bleeding or pseudoaneurysm rupture was initially considered the most likely etiology. However, abdominal CT demonstrated no evidence of intra-abdominal hematoma, suggesting that the bleeding source was unlikely to be located within the abdominal cavity. Endoscopic examination revealed the presence of blood within the jejunum, but no active bleeding was identified at the anastomotic site or within the jejunal mucosa, effectively excluding anastomotic bleeding. Notably, endoscopy also demonstrated that the abdominal drain had migrated into the gastrointestinal tract through the anastomotic leak, providing an anatomical explanation for the external drainage of intraluminal blood. Furthermore, negative findings on DSA largely ruled out intra-abdominal vascular bleeding. The inability of these three conventional diagnostic modalities to localize the source of hemorrhage necessitated a reassessment of its origin. Ultimately, the anesthesiologist's astute observation during endotracheal intubation proved pivotal in establishing the correct diagnosis, underscoring the critical importance of multidisciplinary collaboration in complex clinical scenarios.

Intraluminal migration of a drainage tube into the digestive tract is a rare clinical complication, and its exact incidence remains unclear. Wilmot et al. (8) retrospectively analyzed 254 patients who underwent esophageal surgery and identified four cases (1.6%) in which the drain migrated into the lumen, all occurring in patients with an anastomotic leak, suggesting a close association between the two. However, to our knowledge, no systematic study has specifically addressed drain migration following gastrectomy. Drain migration has been reported to occur either through a pre-existing anastomotic leak or via direct penetration of the intestinal wall resulting from prolonged compression or negative pressure (9). Lai et al. (10) reported a case of drain migration into the esophagojejunal anastomosis after total gastrectomy, with the drain presumably entering through a pre-existing leak. Ravishankar et al. (11) reported a case of spontaneous migration of a drain into the jejunum through a lateral fistula from the Roux loop after total gastrectomy, hypothesizing that collapse and fibrosis of the associated abscess cavity created conditions favorable for migration. Janež et al. (12) reported a case of drain migration into the esophageal lumen after total gastrectomy, with the anastomosis intact and the perforation located 2 cm distal to it, suggesting that local inflammation and persistent compression can also cause migration in the absence of an anastomotic leak. In the present case, the anastomotic leak may have provided a conduit for the drain to enter the digestive tract. With prolonged indwelling, the drain likely migrated into the gastrointestinal lumen under the combined effects of peristalsis and negative pressure. More importantly, the resulting tract functioned as a “short-circuit” pathway for blood drainage: blood originating from posterior nasal bleeding, after being swallowed into the jejunum, was directly evacuated from the body by the negative pressure of the displaced drain, bypassing the normal gastrointestinal transit. This unusual pathway ultimately mimicked the clinical presentation of intra-abdominal hemorrhage.

The etiology in this case was bleeding from the posterior nasal cavity. Compared with anterior epistaxis, posterior nasal bleeding is typically characterized by a larger volume, greater difficulty in achieving hemostasis, a lower likelihood of spontaneous resolution, and a higher risk of airway compromise or aspiration (13). In the present case, the occult nature of posterior nasal bleeding was a key factor contributing to its misdiagnosis as intra-abdominal hemorrhage. Unlike anterior epistaxis, posterior bleeding directs blood into the pharynx and subsequently into the gastrointestinal tract, rather than outward through the nares. The absence of visible nasal bleeding can therefore lead to diagnostic confusion, often resulting in an initial misclassification as upper gastrointestinal bleeding (14). Furthermore, the abdominal drain had migrated through the anastomotic leak into the jejunal lumen, creating an abnormal conduit between the upper gastrointestinal tract and the external environment. Consequently, blood originating from the nasal cavity, after being swallowed, did not present as hematemesis or melena. Instead, it was directly evacuated through the drain, thereby mimicking the clinical features of delayed extra-intestinal hemorrhage. The coexistence of occult posterior nasal bleeding and this aberrant drainage pathway gave rise to the diagnostic challenge observed in this case.

A critical factor in achieving an accurate diagnosis and effective management was the decision not to rely solely on the negative findings of conventional investigations, but to broaden the diagnostic perspective through multidisciplinary collaboration. This experience suggests that in cases of post-gastrectomy hemorrhage with inconclusive results from standard diagnostic modalities, clinicians should maintain a high index of suspicion for uncommon etiologies, including atypical bleeding sources and pathways. Early multidisciplinary consultation is essential to facilitate accurate diagnosis while avoiding unnecessary invasive procedures.

## 4 Conclusion

This report presents the first documented case of a rare bleeding mechanism following gastrectomy, in which hemorrhage originating from the posterior nasal cavity was externally diverted through an abdominal drain that had migrated into the digestive tract via an anastomotic leak, thereby mimicking intra-abdominal bleeding. This case highlights that, in patients presenting with post-gastrectomy hemorrhage in whom contrast-enhanced CT, endoscopy, and angiography fail to localize the bleeding source, a proactive search for remote occult bleeding sites and atypical drainage pathways is warranted. Multidisciplinary collaboration proved essential in establishing the diagnosis, underscoring its critical importance in the management of complex and challenging cases.

## Data Availability

The original contributions presented in the study are included in the article/Supplementary Material, further inquiries can be directed to the corresponding author.
